# Early Attachment to Mothers and Fathers: Contributions to Preschoolers' Emotional Regulation

**DOI:** 10.3389/fpsyg.2021.660866

**Published:** 2021-06-24

**Authors:** Carla Fernandes, Marilia Fernandes, António J. Santos, Marta Antunes, Lígia Monteiro, Brian E. Vaughn, Manuela Verissimo

**Affiliations:** ^1^William James Center for Research, ISPA—Instituto Universitário, Lisbon, Portugal; ^2^Instituto Universitário de Lisboa (ISCTE-IUL), CIS-IUL, Lisboa, Portugal; ^3^Human Development and Family Science, Auburn University, Auburn, AL, United States

**Keywords:** emotion regulation, attachment relationships, mother and father, peer group, preschool

## Abstract

Children acquire and develop emotional regulatory skills in the context of parent-child attachment relationships, nonetheless empirical studies have focused mainly on mother and less information is available regarding the role of both parent-child attachment relationships. Furthermore, despite its importance, there is no information regarding preschool years. This study aims to fill this gap by exploring the potential influences of both mother-child and father-child attachments on preschooler's later emotion regulation observed in the peer group. Fifty-three Portuguese nuclear families (mother, father and focal child) participated in the study; 47% of the children were boys and 53% were girls. Attachment Security was assessed at home using the Attachment Behavior Q-set when children were 3 years of age, and emotion regulation was observed in the preschool classrooms attended by the children at age 5, using the California child Q-sort to derive an Emotion Regulation Q-Scale. Results showed that the combined influence of both parent-child attachment security predicted better emotion regulation results, than did the specific contributions of each parent *per se*. Findings are consistent with integrative approaches that highlight the value of including both mother- and father-child attachment relationships, as well as their combined effect, when studying emotion regulation.

## Introduction

Developmental scientists have long recognized that emotion regulation (ER) is a critical influence on development and competent functioning in childhood (Cole et al., 1994; Saarni, 1999). Hence, assessing its emergence and subsequent organization across age levels is crucial for constructing a developmental account of ER. Self-regulation processes do not occur in a vacuum, and early relational experiences are thought to shape and guide these processes during childhood (e.g., Goldberg, 2000; Birmingham et al., 2017). With respect to children's ER abilities, most empirical evidence centers on mothers' contributions, with less studies on fathers' contributions, and with even fewer considering both parents (for reviews see Zimmer-Gembeck et al., 2015; Cooke et al., 2019). To our knowledge, no study examining the association between both parent-child attachments and emotional regulation has focused on preschool years. Thus, the present study aims to contribute to bridging this gap by analyzing the independent and joint contributions of early relational experiences with both mothers and fathers at the beginning of the pre-school years (age 3) to children's emotional regulation at the end of this period (age 5).

Emotion regulation is a complex concept with multiple definitions (Brumariu, 2015). In this study, it was conceptualized as the individual's ability to effectively modulate emotional arousal in order to achieve optimal levels of engagement with the environment (Cicchetti et al., 1991; Thompson, 1994). According to Shields and Cicchetti (1997), ER reflects differences in lability, flexibility, and situational responsiveness that allows for appropriate emotional expression in emotionally challenging situations and promotes adaptive functioning. Regulation capacities emerge as a result of the interplay between biological and social processes (e.g., Kidwell and Barnett, 2007), which include the quality of family relationships. There is a broad consensus among researchers that the quality of early attachment relationships plays a prominent role (Thompson, 1991; Calkins and Hill, 2007; Thompson and Meyer, 2007; Brumariu, 2015) that grounds development of ER in early childhood (Cooke et al., 2019). Thus, ER can be seen (in part) as a developmental consequence of earlier attachment relationships (e.g., Kerns, 2008).

Attachment theory highlights the self-regulatory capacities fostered by the child's use of the parent as a secure base for exploring the environment, and on the safety-regulatory capacities when the child is able to activate the parent as a safe haven to return, when the child desires contact or needs assistance (Ainsworth et al., 1978; Bowlby, 1982, 1988; Waters and Cummi1ngs, 2000). Sroufe and Waters (1977) proposed that in the first year of life, the attachment figure serves as the primary source of regulation for affect (soothing, arousing when useful) and modulates the child's experiences of affect fluctuations. Around 24–36 months develops a partnership between the attachment figures and the child that jointly regulate emotions, and after 48 months children are beginning to self-regulate emotion even when the attachment figure is not present.

Bowlby (1982, 1988) suggested that qualitatively different patterns of emotional response and self-regulation could emerge from different parent-child attachment histories characterized by the caregivers' responsiveness to their infants' distress in everyday interactions. For example, in secure attachments relationships, caregivers tend to be more aware of and responsive to children's feelings (both positive and negative) and they are more available to engage in conversations about those feelings. By contrast caregivers of children with insecure attachments tend to be less (or less consistently) responsive to their children's feelings, and less likely to engage in conversations to help them dealing with their difficult emotional experiences (Cassidy, 1994; Goldberg et al., 1994; Thompson, 1994; Thompson et al., 2003).

So, in the context of secure attachment, children are able to co-construct an enduring emotional security and have opportunities to effectively co-regulate distress. Several empirical studies support theoretical assumptions (Morris et al., 2007; Zimmer-Gembeck et al., 2015; for reviews see Cooke et al., 2019). Children with secure attachments, when compared with children with insecure ones, are expected to be advantaged regarding the acquisition of effective emotion regulatory capacities (Thompson and Meyer, 2007; Brumariu, 2015; Zimmer-Gembeck et al., 2015). Overall, findings show that secure children are more likely to be emotionally competent in terms of expression, emotional knowledge, emotional flexibility, and appropriate affect regulation, when contrasted to children with insecure attachments (e.g., Kochanska, 2001; Denham et al., 2002; Calkins and Hill, 2007; Kerns et al., 2007; Brumariu et al., 2012; Roque et al., 2013).

Evidence also suggests that, in the context of secure parent-child attachment relationships, children learn and internalize effective ER capacities/strategies and use them across time and situations where attachment figures may not be present (e.g., in the peer group, Sroufe, 1983; Contreras and Kerns, 2000; Brumariu, 2015; Zimmer-Gembeck et al., 2015). Findings from the National Institute of Child Health and Human Development (NICHD) Study of Early Child Care and Youth Development (NICHD Early Child Care Research Network., 2005) also provides evidence for the association between early attachment and later preschoolers' self-regulation, in terms of their self-control, attentional impulsivity, and engagement in school settings (Drake et al., 2014).

Over the last few years there has been an effort to include the father in attachment research (Ahnert and Schoppe-Sullivan, 2019; Cowan and Cowan, 2019), based on theoretical and empirical expansions designed to include attachment figures beyond the mother (e.g., Suess et al., 1992; van Ijzendoorn, 2005; Monteiro et al., 2010; Dagan and Sagi-Schwartz, 2018; Grossmann and Grossmann, 2019). Studies that have included both attachment figures provide findings consistent with the notions that each attachment relationship is independently co-constructed between the parent and child; that children typically use both mothers and fathers as a secure base; and the contexts and interactional patterns between the child and each of the caregivers may be distinct and unique (e.g., Bowlby, 1982; Grossmann et al., 2002; Monteiro et al., 2008, 2010; Kochanska and Kim, 2013). It is presumed that the interactive style characteristic of the mother-child relationships is mostly directed to calm, reassure and soothing the child, contrasting with the interactive nature of father-child relationships mainly associated with more emotional arousal, higher levels of excitation or destabilization (Paquette, 2004). Qualitatively different styles of interaction that lead to the formation and maintenance of each attachment relationship may translate into a specific impact on children's developmental trajectories (Tamis-LeMonda, 2004; Booth-Laforce et al., 2006; Veríssimo et al., 2011). At this level, evidence suggest the possibility of attachment to each parent support distinct spheres of influence on children's development (e.g., Veríssimo et al., 2011), but also that there may be interactive influences of the two relationships for other outcome domains (e.g., Dagan and Sagi-Schwartz, 2018; Fernandes et al., 2020). From an integrative perspective, stronger predictions of children's developmental outcomes could be obtained from joint effects of mother–child and father–child attachment relationships, than when considering their influences separately (e.g., van Ijzendoorn, 2005; Dagan and Sagi-Schwartz, 2018).

Nonetheless when looking at the studies relating attachment and ER, the majority have focused primarily on mother–child attachment relationships and less information is available regarding father's role (Morris et al., 2007). In a recent meta-analysis (Cooke et al., 2019), that examined the associations between parent–child attachment and emotion domains, only 16 studies (from a total of 72) included both mother and father data. The majority of these studies (*n* = 14) focused on later ages (9–18 years), with fewer studies (*n* = 2) in early ages (12–13 months). At this level, early childhood has been understudied, lacking information, for example, regarding preschool years.

Because fathers have been understudied in both attachment and ER research and more longitudinal research is needed, questions remain in terms of the unique contributions of each caregiver, as well as the (possible) joint effects on children's ER. Such data will contribute to the current state of knowledge, adding to the literature that has started to focus on multiple attachment figures (e.g., van Ijzendoorn, 2005; Dagan and Sagi-Schwartz, 2018; Cowan and Cowan, 2019; Grossmann and Grossmann, 2019; Ahnert and Schoppe-Sullivan, 2019). Furthermore, it will help to fill the gap of information regarding preschool years. This period is thought to be critical in the development of ER (e.g., Sala et al., 2014), since children are becoming increasingly more autonomous in their regulation of emotions, and need less adult support (e.g., Cole and Hall, 2008). Outside the family, (pre)school is the first context where ER skills can be observed in peer groups, in the absence of parental figures (e.g., Sala et al., 2014).

Thus, the aim of this study was to examine potential influences of both parent–child attachments (when children were on average 3 years of age) on later children's ER (assessed two years later). We used behavior-relevant observations to assess both security of attachment and ER in ecological valid contexts of children's lives (e.g., family and preschool settings, respectively).

## Methods

### Participants

Fifty-three Portuguese nuclear families (mother, father, and focal child), with both parents living in the household, participated in the study. Mother–child and father–child attachment relationships were observed when children were 3 years of age (*M* = 36.87 months; *SD* = 6.91), and Emotion Regulation was assessed when children were 5 years (*M* = 68.97 months; *SD* = 3.95), 47% of the children were boys and 53% were girls. Children's age of first school entry was in average 11.35 months (SD = 10.59). Fifty-five percent of the mothers and 59% of fathers reported having a university degree with the remaining having high-school education, 96% of mothers and 98% of fathers worked full-time. Families were middle class by the standards of the local community.

### Instruments and Procedures

This study is part of an ongoing research project (for previous related work see Veríssimo et al., 2011; Fernandes et al., 2020), approved by the Ethics committee of the ISPA—University Institute. Informed consents were obtained from all the participating families when they were recruited to the study. No families had more than one child in the relevant age range when recruitment began. Imposed exclusion criteria for being included in this study were if a child is diagnosed for mental delay or for a physical diagnosis (e.g., blind, Down syndrome, etc.).

*Attachment Security* was assessed at home using the Attachment Behavior Q-set (AQS, Waters, 1995, v. 3.0) when children were 3 years of age. The AQS evaluates the organization of children's secure base behavior in an ecological valid context and is especially valuable when mothers and fathers are assessed.

Mother–Child and father–child dyads were observed during one home visit each, lasting between 2 and 3 h, and with ~1-month interval. The visits were counterbalanced and just one of the parents was present at the home. Parents were informed that the main objective of the visit was to study child parent interaction and were asked to maintain their daily routines as if observers were not present. Different pairs of observers conducted the home visits, with the two observers for the mother being different from those of the father. Observers were trained for a period of several weeks before initiating formal observations for the project. After training, inter-observer agreement (Q-correlation) was between 0.72 and 0.92. Rater agreements during actual data collection averaged 0.77 for the mothers and 0.79 for the fathers. The final Q-sort for the child was a composite (average) of the two Q-descriptions provided by each observer and criterion scores for security were calculated using this composite. After the visit, observers independently complete the AQS by allocating the 90 items into nine categories, according to a fixed distribution. For scoring, the Q-description of the observed child was compared to the “security criterion sort” described by Waters (1995).

*Emotion Regulation (ER)* data was collected in preschool classrooms when children were 5 years. Distinct teams of two independent observers, spent 20 h in each classroom, observing the group in different moments and activities. Each observer described each child independently, using the California child Q-sort (CCQ; Block and Block, 1980) intended to capture children's behavior profiles, through a nine-category rectangular distribution. The median of intra-class correlation estimated for each pair of observers in each classroom was.93. The final CCQ for the child was a composite (average) of the two Q-descriptions provided by each observer. *Emotion Regulation Q-Scale* (Shields and Cicchetti, 1997) was derived from CCQ. This Q-scale was developed by experts and describes the personality profile of an optimally well-regulated child, defining ER in terms of lability, flexibility, and modulation of one's emotions. In the original study (Shields and Cicchetti, 1997), the construct validity was established through comparisons with additional teacher checklists and observations. Internal consistency as assessed through Cronbach's a was reported as 0.98 (Shields and Cicchetti, 1997).

### Plan of Analyses

Before our main analyses, descriptive statistics were explored. Differences between boys and girls were tested using independent *t*-tests. Associations and mean differences between mother–child (MS) and father–child (FS) attachment security were also tested, using Pearson's correlation coefficient and paired *t*-test, respectively. Associations between attachment security and emotion regulation (ER) were explored using Pearson's correlation coefficient. Next, we tested for the predictive value MS and FS on children's ER using regression analyses. How MS and FS interact in their influence on children's ER was also explored. Significance of the interaction term was analyzed using PROCESS macro v3.5 for SPSS (Hayes, 2018), with bootstrapping (95% CI; 5,000 samples). As recommended by Dearing and Hamilton (2006), graphic representations were supplemented by analyzing and regions of significance (i.e., Johnson-Neyman technique; Johnson and Neyman, 1936).

## Results

Descriptive statistics are presented in Table 1. No significant differences between boys and girls were found for either mother–child (MS) or father–child attachment security, neither for emotion regulation (ER).

**Table 1:** Means and standard deviations for attachment security and emotion regulation.

	**Attachment**		**Emotion regulation**
	**Mother**	**Father**	
Total sample	0.50 (0.20)	0.51 (0.19)	7.22 (0.67)
Boys	0.53 (0.19)	0.55 (0.14)	7.40 (0.49)
Girls	0.48 (0.22)	0.47 (0.23)	7.06 (0.76)

Attachment security scores were within the range of typical values for a non-clinical sample as ours (van IJzendoorn et al., 2004). There was a significant positive correlation between MS and FS (*r* = 0.41; *p* < 0.01) and the mean difference between them was not significant [*t*_(52)_ = −0.19; *p* = 0.85].

Regression analysis tested for the main effects of MS and FS on ER. A significant effect was found for FS (*B* = 0.27, *t* = 2.02, and *p* = 0.05) but not for MS (*B* = 0.17, *t* = 1.24, and *p* = 0.22).

**Figure 1:**
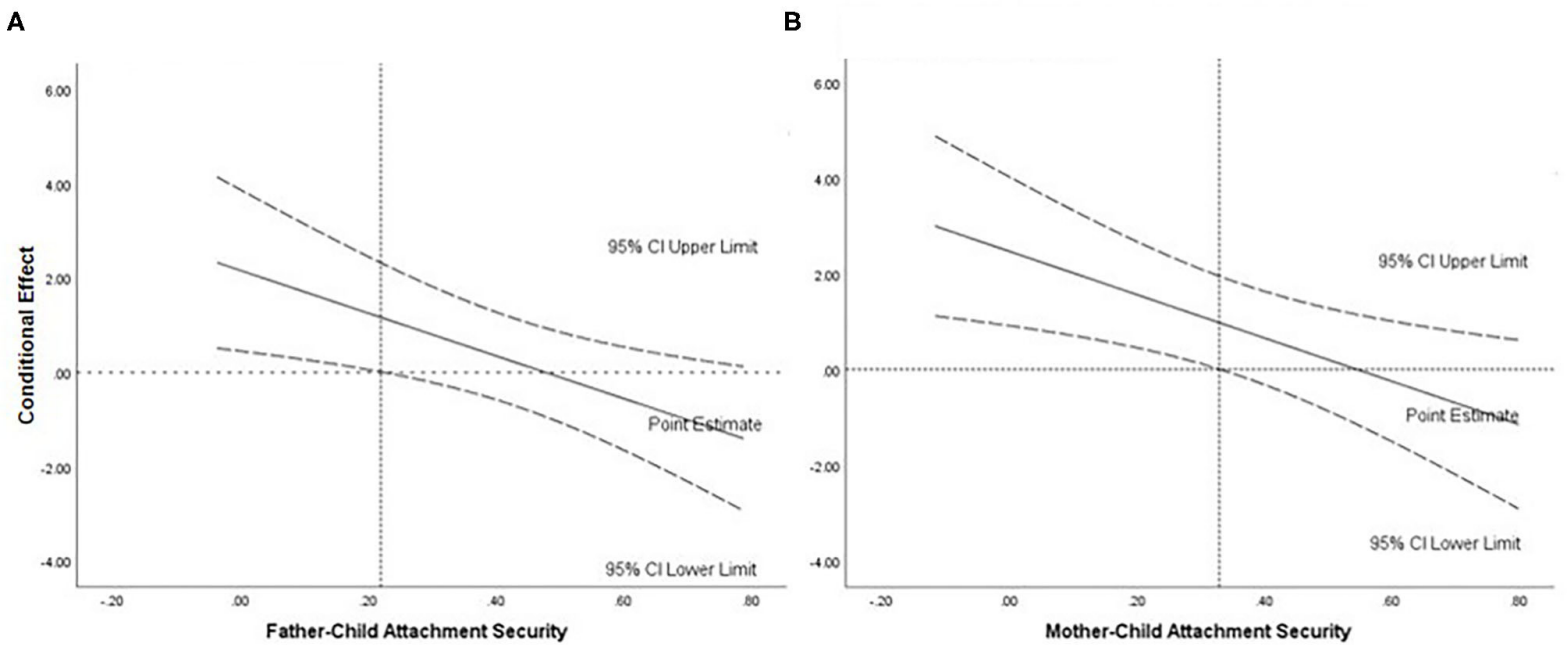
Visual representation of the interaction of parent–child attachment security on emotion regulation. **(A)** Conditional effect of mother security on emotional regulation as a function of father security. **(B)** Conditional effect of father security on emotional regulation as a function of mother security.

In the following analysis we tested how MS and FS might interact in their influence on children's ER, for that we used the PROCESS macro for SPSS. The interaction term (product of MS and FS) was a significant predictor of ER (*b* = – 4.53, 95% CI [−7.92, −1.15], *t* = −2.70, *p* < 0.01) and increased the explained variance by 12% [*R*^2^ = 0.20; *F*_(1, 49)_ = 7.26; *p* < 0.01]. First, we used father as moderator and when we analyzed regions of significance using Johnson–Neyman technique (see Figure 1A) we identify that when FS is lower or equal to 0.22 the conditional effect of MS is positive and significantly different from zero (*p* < 0.05). Meaning that for those children with lower father–child attachment security the model estimates higher ER as the mother–child attachment security gets higher. We performed the same analysis using the mother's score as the moderator and identified that for MS the estimated point below which the conditional effect was significant was 0.33, *p* < 0.05 (see Figure 1B). Again, the effect was positive, meaning that for those children with lower mother-child attachment security the model estimates higher ER as the father-child attachment security gets higher.

## Discussion

This study was designed to explore the potential influences of both parent–child attachments assessed early in the preschool period on ER (e.g., lability, flexibility, situational responsivity, and modulation of one's emotional arousal) observed in peer groups, at the end of preschool. Counter to our expectation, we did not find a significant association between mother-child attachment security and ER. We did find, however, that father–child attachment security significantly predicted child ER. This may have to do with the way emotional regulation was conceptualized and measured in the present study. The Shields and Cicchetti (1997) definition of ER reflects the differences in emotionally arousing situations. Father–child interactions tend to be characterized by greater emotional arousal and more unpredictability, providing greater opportunities for learning emotion regulatory skills within the context of these exchanges (Parke, 1996; Paquette, 2004). Specifically, with respect to interactive physical play, it has been postulated that the unique nature of father-child interactions fosters the gaining and development of adaptive regulatory abilities, later mobilized and displayed in peer interactions (Lieberman et al., 1999; Coleman, 2003; Booth-Laforce et al., 2006; McDowell and Parke, 2009; Lindsey et al., 2010; Chae and Lee, 2011). Fathers are expected to stand out in terms of active play with their children, and evidence shows that children may benefit from regular and moderate levels of father-child active physical play, achieving better developmental outcomes (Bocknek et al., 2017; Amodia-Bidakowska et al., 2020). It might be that, in the context of active play with their fathers, children experience intense emotions in a safe and controlled manner, learning how to better regulate them. Furthermore, qualitatively different interaction styles may reflect different parental emotion socialization strategies, fostering different emotional developmental outcomes. Mother–child interactions are more likely to foster children's emotional understanding whereas father interaction more likely to foster stronger emotion regulatory skills (McDowell et al., 2002; Brumariu, 2015).

Perhaps more importantly, our results suggest that the combined influences of attachments to each parent provide a stronger prediction to children's ER in the preschool (than do either parent alone), which is consistent with an integrative approach (e.g., van Ijzendoorn, 2005; Dagan and Sagi-Schwartz, 2018). Particularly, these interaction results suggest that for children with low security scores to one parent, it is beneficial to have a secure relationship with the other parent, in the sense that the two relationships interact to predict better ER in the peer group. These findings are consistent with previous findings suggesting that attachment security fosters ER, with securely attached children being more likely to display more effective ER skills (Morris et al., 2007; Thompson and Meyer, 2007; Zimmer-Gembeck et al., 2015; Cooke et al., 2019). Moreover, children use these skills across time and situations outside the family context (e.g., Contreras and Kerns, 2000; Drake et al., 2014; Brumariu, 2015). It will be important that future research continue to consider the joint influences of both parent-child attachment relationships on children's developmental outcomes (e.g., emotion regulation), mirroring life contexts where children are raised (Dagan and Sagi-Schwartz, 2018), including all family typologies (i.e., traditional, and non-traditional).

Our findings suggest the possibility that a secure attachment with one parent could protect against the risk for difficulties in children's emotion regulatory capacities displayed in peer groups, buffering the impact of a less secure attachment with the other parent. In this sense, it may be the case that children securely attached to only one parent to achieve ER outcomes comparable to those of children securely attached to both parents (Dagan and Sagi-Schwartz, 2018). Integrating these findings with previous ones on later ages (e.g., Piermattei et al., 2017; Pace et al., 2018; Rogier et al., 2020) could help to better understand the role that parent-child attachment relationships and their impact on children's emotion regulatory capacities play on later functioning, well-being and psychopathology, since it is presumed that these associations in adolescence and adulthood are built on a history of early attachment relationships (Brumariu, 2015).

We also recognize limitations that constrain the generalization of these results. For instance, we do not have ER data at 3 years of age (i.e., at Time 1) for this sample, however, it would have been important to control this covariate when testing the predictive role of attachment. Given the small size and lack of heterogeneity of the present sample, it would be important to replicate these findings in larger non-convenience samples. For instance, using *G Power Software* (version 3.1.9.4) (Faul et al., 2007), a power analyses, with an alpha = 0.05 and power = 0.80, showed that the minimum sample size needed to detect an effect size of *f* = 0.15 would be *n* = 77 (for a linear multiple regression: fixed model, 3 predictors).

Also, in order to enhance the predictive power of infant attachment relationships on ER outcomes, it would be useful to replicate this study in clinical and risk samples, where the probability of less secure attachment is higher. In this sense, findings of the present study are preliminary and, although caution is needed regarding interpretations, could represent hypotheses for future research. Overall, findings of this study shed light to the importance of including both mother– and father–child attachment relationships and considering their combined influences when studying emotion regulation, to enhance research on this topic.

## Data Availability Statement

The raw data supporting the conclusions of this article will be made available by the authors, without undue reservation.

## Ethics Statement

The studies involving human participants were reviewed and approved by ISPA Ethics Committee. Written informed consent to participate in this study was provided by the participants' legal guardian/next of kin.

## Author Contributions

MV, AS, and CF: conception of the work. CF, MF, MA, and LM: data collection. CF, MF, MV, and AS: data analysis and drafting the manuscript. CF, MF, MV, AS, LM, and BV: data interpretation and edit the manuscript. All authors read and commented on the manuscript.

## Conflict of Interest

The authors declare that the research was conducted in the absence of any commercial or financial relationships that could be construed as a potential conflict of interest.

## References

[B1] Ahnert L. Schoppe-Sullivan S. (2019). Fathers from an attachment perspective. Attach. Hum. Dev. 22, 1–3. 10.1080/14616734.2019.1589054 30982424

[B2] Ainsworth M. D. S. Blehar M. C. Waters E. Wall S. (1978). Patterns of Attachment: A Psychological Study of the Strange Situation. Lawrence Erlbaum.

[B3] Amodia-Bidakowska A. Laverty C. Ramchandani P. (2020). Father-child play: a systematic review of its frequency, characteristics and potential impact on children's development. Dev. Rev. 57:100924. 10.1016/j.dr.2020.100924

[B4] Birmingham R. S. Bub K. L. Vaughn B. E. (2017). Parenting in infancy and self-regulation in preschool: an investigation of the role of attachment history. Attach. Hum. Dev. 19, 107–129. 10.1080/14616734.2016.1259335 27894211 PMC5592094

[B5] Block J. H. Block J. (1980). The California Child Q-Set. Palo Alto, CA: Consulting Psychologists.

[B6] Bocknek E. L. Dayton C. Raveau H. A. Richardson P. Brophy-Herb H. E. Fitzgerald H. E. (2017). Routine active playtime with fathers is associated with self-regulation in early childhood. Merrill Palmer Q., 63, 105–134. 10.13110/merrpalmquar1982.63.1.0105

[B7] Booth-Laforce C. Oh W. Kim A. H. Rubin K. H. Rose-Krasnor L. Burgess K. (2006). Attachment, self-worth, and peer-group functioning in middle childhood. Attach. Hum. Dev. 8, 309–325. 10.1080/14616730601048209 17178610

[B8] Bowlby J. (1982). Attachment and Loss: Vol. 1 Attachment. New York, NY: Basic Books (Original work published in 1969).

[B9] Bowlby J. (1988). A Secure Base: Parent-Child Attachment and Healthy Human Development. New York, NY: Basic.

[B10] Brumariu L. E. (2015). “Parent-child attachment and emotion regulation,” in Attachment in Middle Childhood: Theoretical Advances and New Directions in an Emerging Field, eds Bosman G. Kerns K. A. (Wiley Periodicals Inc.), 31–45. 10.1002/cad.20098 26086126

[B11] Brumariu L. E. Kerns K. A. Seibert A. C. (2012). Mother-child attachment, emotion regulation, and anxiety symptoms in middle childhood. Pers. Relationsh. 19, 569–585. 10.1111/j.1475-6811.2011.01379.x

[B12] Calkins S. D. Hill A. (2007). “Caregiver influences on emerging emotion regulation: biological and environmental transactions in early development,” in Handbook of Emotion Regulation, ed Gross J. J. (New York, NY: The Guilford Press), 229–248.

[B13] Cassidy J. (1994). “Emotion regulation: influences on attachment relationships,” in The Development of Emotion Regulation: Biological and Behavioral Considerations. Monographs of the Society for Research in Child Development, Vol. 59, ed Fox N. A. (Chicago: University of Chicago), 228–249. 7984163

[B14] Chae J. Lee K. (2011). Impacts of Korean fathers' attachment and parenting behavior on their children's social competence. Soc. Behav. Pers. Int. J. 39, 627–643. 10.2224/sbp.2011.39.5.627

[B15] Cicchetti D. Ganiban J. Barnett D. (1991). “Contributions from the study of high-risk populations to understanding the development of emotion regulation,” in Cambridge Studies in Social and Emotional Development. The Development of Emotion Regulation and Dysregulation, eds Garber J. Dodge K. A. (Cambridge: Cambridge University Press), 15–48.

[B16] Cole P. M. Hall S. E. (2008). “Emotion dysregulation as a risk factor for psychopathology,” in Child and Adolescent Psychopathology, eds Beauchaine T. P. Hinshaw S. P. (JohnWiley and Sons), 265–298.

[B17] Cole P. M. Michel M. K. Teti L. O. (1994). The development of emotion regulation and dysregulation: A clinical perspective. Monogr. Soc. Res. Child Dev. 59, 73–100, 250–283. 10.2307/1166139 7984169

[B18] Coleman P. K. (2003). Perceptions of parent-child attachment, social self-efficacy, and peer relationships in middle childhood. Infant Child Dev. 12, 351–368. 10.1002/icd.316

[B19] Contreras J. M. Kerns K. A. (2000). “Emotion regulation processes: explaining links between parent-child attachment and peer relationships,” in Family and Peers: Linking Two SocialWorlds, eds Kerns K. A. Contreras J. M. Neal-Barnett A. M. (Westport: Praeger), 1–25.

[B20] Cooke J. Kochendorfer L. Stuart-Parrigon K. Koehn A. Kerns K. (2019). Parent-child attachment and children's experience and regulation of emotion: a meta-analytic review. Emotion 19, 1103–1126. 10.1037/emo0000504 30234329

[B21] Cowan P. Cowan C. (2019). The role of parental relationships in children's well-being: a modest set of proposals for improving the lives of children. Hum. Dev. 62, 171–174. 10.1159/000500173

[B22] Dagan O. Sagi-Schwartz A. (2018). Early attachment network with mother and father: an unsettled issue. Child Dev. Perspect. 12, 115–121. 10.1111/cdep.12272

[B23] Dearing E. Hamilton L. C. (2006). Best practices in quantitative methods for developmentalists: V. Contemporary advances and classic advice for analyzing mediating and moderating variables. Monogr. Soc. Res. Child Dev. 71, 88–104. 10.1111/j.1540-5834.2006.00406.x 17199773

[B24] Denham S. A. Blair K. Schmidt M. DeMulder E. (2002). Compromised emotional competence: Seeds of violence sown early? Am. J. Orthopsychiatry 72, 70–82. 10.1037/0002-9432.72.1.70 14964596

[B25] Drake K. Belsky J. Fearon R. M. (2014). From early attachment to engagement with learning in school: the role of self-regulation and persistence. Dev. Psychol. 50, 1350–1361. 10.1037/a0032779 23647414

[B26] Faul F. Erdfelder E. Lang A.-G. Buchner A. (2007). G^*^Power 3: a flexible statistical power analysis program for the social, behavioral, and biomedical sciences. Behav. Res. Methods 39, 175–191. 10.3758/BF03193146 17695343

[B27] Fernandes C. Monteiro L. Santos A. J. Fernandes M. Antunes M. Vaughn B. E.. (2020). Early father-child and mother-child attachment relationships: Contributions to preschoolers' social competence. Attach Hum Dev. 22, 687–704. 10.1080/14616734.2019.1692045 31739746

[B28] Goldberg S. (2000). Attachment and Development. London: Hodder Arnold.

[B29] Goldberg S. MacKay-Soroka S. Rochester M. (1994). Affect, attachment, and maternal responsiveness. Infant Behav. Dev. 17, 335–339. 10.1016/0163-6383(94)90013-2

[B30] Grossmann K. Grossmann K. E. (2019): Essentials when studying child-father attachment: a fundamental view on safe haven secure base phenomena. Attach. Hum. Dev. 22, 9–14. 10.1080/14616734.2019.1589056 30898025

[B31] Grossmann K. Grossmann K. E. Fremmer-Bombik E. Kindler H. Scheuerer-Englisch H. Zimmerman P. (2002). The uniqueness of the child-father attachment relationship: fathers' sensitive and challenging play as a pivotal variable in a 16-year longitudinal study. Soc. Dev. 11, 307–331. 10.1111/1467-9507.00202

[B32] Hayes A. F. (2018). Introduction to Mediation, Moderation, and Conditional Process Analysis. New York, NY: Guildford Press.

[B33] Johnson P. O. Neyman J. (1936). Tests of certain linear hypotheses and their application to some educational problems. Stat. Res. Memoirs 1, 57–93.

[B34] Kerns K. A. (2008). “Attachment in middle childhood,” in Handbook of Attachment: Theory, Research, and Clinical Applications, eds Cassidy J. Shaver P. R. (New York, NY: The Guilford Press), 366–382.

[B35] Kerns K. A. Abraham M. Schlegelmilch A. Morgan T. (2007). Mother-child attachment in later middle childhood: Assessment approaches and associations with mood and emotion regulation. Attach. Hum. Dev. 9, 33–53. 10.1080/14616730601151441 17364481

[B36] Kidwell S. L. Barnett D. (2007). Adaptive emotion regulation among low-income African American children. Merrill Palmer Q. 53, 155–183. 10.1353/mpq.2007.0011

[B37] Kochanska G. (2001). Emotional development in children with different attachment histories: the first three years. Child Dev. 72, 474–490. 10.1111/1467-8624.00291 11333079

[B38] Kochanska G. Kim S. (2013). Early attachment organization with both parents and future behaviour problems: from infancy to middle childhood. Child Dev. 84, 283–296. 10.1111/j.1467-8624.2012.01852.x 23005703 PMC3530645

[B39] Lieberman M. Doyle A. B. Markiewicz D. (1999). Developmental patterns in security of attachment to mother and father in late childhood and early adolescence: associations with peer relations. Child Dev. 70, 202–213. 10.1111/1467-8624.00015 10191523

[B40] Lindsey E. W. Cremeens P. R. Caldera Y. M. (2010). Mother-child and father-child mutuality in two contexts: consequences for young children's peer relationships. Infant Child Dev. 19, 142–160. 10.1002/icd.645

[B41] McDowell D. J. Kim M. O'Neil R. Parke R. D. (2002). “Children's emotional regulation and social competence in middle childhood: the role of maternal and paternal interactive style,” in Emotions and the Family, ed Fabes R. A. (Philadelphia, PA: The Haworth Press, Inc.), 345–364.

[B42] McDowell D. J. Parke R. D. (2009). Parental correlates of children's peer relations: an empirical test of a tripartite model. Dev. Psychol. 45, 224–235. 10.1037/a0014305 19210004

[B43] Monteiro L. Veríssimo M. Vaughn B. E. Santos A. J. Bost K. B. (2008). Secure base representations for both fathers and mothers predict children's secure base behavior in a sample of Portuguese families. Attach. Hum. Dev. 10, 189–206. 10.1080/14616730802113711 18773318

[B44] Monteiro L. Verissimo M. Vaughn B. E. Santos A. J. Torres N. Fernandes M. (2010). The organization of children's secure base behaviour in two-parent Portuguese families and father's participation in child-related activities. Eur. J. Dev. Psychol. 7, 545–560. 10.1080/17405620902823855

[B45] Morris A. Silk J. Steinberg L. Myers S. Robinson L. (2007). The role of the family context in the development of emotion regulation. Soc. Dev. 16, 361–388. 10.1111/j.1467-9507.2007.00389.x 19756175 PMC2743505

[B46] NICHD Early Child Care Research Network (2005). Child Care and Child Development: Results from the NICHD Study of Early Child Care and Youth Development. New York, NY: The Guilford Press.

[B47] Pace C. S. Di Folco S. Guerriero V. (2018). Late-adoptions in adolescence: can attachment and emotion regulation influence behaviour problems? A controlled study using a moderation approach. Clin. Psychol. Psychother. 25, 250–262. 10.1002/cpp.2158 29193445

[B48] Paquette D. (2004). Theorizing the father-child relationship: mechanisms and developmental outcomes. Hum. Dev. 47, 193–129. 10.1159/000078723

[B49] Parke R. D. (1996). The Developing Child Series. Fatherhood. Harvard University Press.

[B50] Piermattei C. Pace C. S. Tambelli R. D'Onofrio E. Di Folco S. (2017). Late adoptions: attachment security and emotional availability in mother-child and father-child dyads. J. Child Fam. Stud. 26, 2114–2125. 10.1007/s10826-017-0732-6

[B51] Rogier G. Beomonte Zobel S. Velotti P. (2020). Pathological personality facets and emotion (dys)regulation in gambling disorder. Scand. J. Psychol. 61, 262–270. 10.1111/sjop.12579 31625173

[B52] Roque L. Veríssimo M. Fernandes M. Rebelo A. (2013). Emotion regulation and attachment: relationships with children's secure base, during different situational and social contexts in naturalistic settings. Infant Behav. Dev. 36, 298–306. 10.1016/j.infbeh.2013.03.003 23542812

[B53] Saarni C. (1999). The Development of Emotional Competence. New York, NY: Guilford Press.

[B54] Sala M. N. Pons F. Molina P. (2014). Emotion regulation strategies in preschool children. Br. J. Dev. Psychol. 32, 440–453. 10.1111/bjdp.12055 25040163

[B55] Shields A. Cicchetti D. (1997). Emotion regulation among school-age children: The development and validation of a new criterion Q-sort scale. Dev. Psychol. 6, 906–916. 10.1037/0012-1649.33.6.906 9383613

[B56] Sroufe L. A. (1983). “Infant-caregiver attachment and patterns of adaptation in preschool: the roots of maladaptation and competence,” in Minnesota Symposium on Child Psychology: Vol. 16, Development and Policy Concerning ChildrenWith Special Needs, ed Perlmutter M. (Erlbaum), 41–83.

[B57] Sroufe L. A. Waters E. (1977). Attachment as an organizational construct. Child Dev. 48, 1184–1199. 10.2307/1128475

[B58] Suess G. J. Grossmann K. E. Sroufe L. A. (1992). Effects of infant attachment to mother and father on quality of adaptation in preschool: from dyadic to individual organization of self. Int. J. Behav. Dev. 15, 43–65. 10.1177/016502549201500103

[B59] Tamis-LeMonda C. S. (2004). Conceptualizing father's roles: playmates and more. Hum. Dev. 47, 220–227. 10.1159/000078724

[B60] Thompson R. A. (1991). Emotional regulation and emotional development. Educ. Psychol. Rev. 3, 269–307. 10.1007/BF01319934

[B61] Thompson R. A. (1994). Emotion regulation: a theme in search of definition. Monogr. Soc. Res. Child Dev. 59, 25–52. 10.2307/1166137 7984164

[B62] Thompson R. A. Laible D. J. Ontai L. L. (2003). “Early understandings of emotion, morality, and self: developing a working model,” in Advances in Child Development and Behavior, Vol. 31, ed Kail R. V. (Cambridge: Academic Press), 137–171. 10.1016/s0065-2407(03)31004-3 14528661

[B63] Thompson R. A. Meyer S. (2007). “Socialization of emotion regulation in the family,” in Handbook of Emotion Regulation, ed Gross J. J. (New York: Guilford Press), 249–268.

[B64] van Ijzendoorn M. (2005). Attachment in social networks: toward an evolutionary social network model. Hum. Dev. 48, 85–88. 10.1159/000083218

[B65] van IJzendoorn M. H. Vereijken C. M. Bakermans-Kranenburg M. J. Riksen-Walraven J. M. (2004). Assessing attachment security with the attachment Q-sort: meta-analytic evidence for the validity of the observer AQS. Child Dev. 75, 1188–1213. 10.1111/j.1467-8624.2004.00733.x 15260872

[B66] Veríssimo M. Santos A. J. Vaughn B. E. Torres T. Monteiro L. Santos O. (2011). Quality of attachment to father and mother and number of reciprocal friends. Early Child Dev. Care 181, 27–38. 10.1080/03004430903211208

[B67] Waters E. (1995). Appendix A: attachment Q-set (version 3.0). Monogr. Child Dev. 60, 234–246. 10.1111/j.1540-5834.1995.tb00214.x

[B68] Waters E. Cummi1ngs E. M. (2000). A secure base from which to explore close relationships. Child Dev. 71, 164–172. 10.1111/1467-8624.00130 10836570

[B69] Zimmer-Gembeck M. J. Webb H. J. Pepping C. A. Swan K. Merlo O. Skinner E. A.. (2015). Review: is parent-child attachment a correlate of children's emotion regulation and coping? Int. J. Behav. Dev. 41, 74–93. 10.1177/0165025415618276

